# Mitotic Illegitimate Recombination Is a Mechanism for Novel Changes in High-Molecular-Weight Glutenin Subunits in Wheat-Rye Hybrids

**DOI:** 10.1371/journal.pone.0023511

**Published:** 2011-08-22

**Authors:** Zhongwei Yuan, Dengcai Liu, Lianquan Zhang, Li Zhang, Wenjie Chen, Zehong Yan, Youliang Zheng, Huaigang Zhang, Yang Yen

**Affiliations:** 1 Triticeae Research Institute, Sichuan Agricultural University at Chengdu, Wenjiang, Sichuan, People's Republic of China; 2 Key Laboratory of Adaptation and Evolution of Plateau Biota, Northwest Institute of Plateau Biology, Chinese Academy of Sciences, Xining, People's Republic of China; 3 Department of Biology and Microbiology, South Dakota State University, Brookings, South Dakota, United States of America; National Cancer Institute, United States of America

## Abstract

Wide hybrids can have novel traits or changed expression of a quantitative trait that their parents do not have. These phenomena have long been noticed, yet the mechanisms are poorly understood. High-molecular-weight glutenin subunits (HMW-GS) are seed storage proteins encoded by *Glu-1* genes that only express in endosperm in wheat and its related species. Novel HMW-GS compositions have been observed in their hybrids. This research elucidated the molecular mechanisms by investigating the causative factors of novel HMW-GS changes in wheat-rye hybrids. HMW-GS compositions in the endosperm and their coding sequences in the leaves of F_1_ and F_2_ hybrids between wheat landrace Shinchunaga and rye landrace Qinling were investigated. Missing and/or additional novel HMW-GSs were observed in the endosperm of 0.5% of the 2078 F_1_ and 22% of 36 F_2_ hybrid seeds. The wildtype *Glu-1Ax* null allele was found to have 42 types of short repeat sequences of 3-60 bp long that appeared 2 to 100 times. It also has an in-frame stop codon in the central repetitive region. Analyzing cloned allele sequences of HMW-GS coding gene *Glu-1* revealed that deletions involving the in-frame stop codon had happened, resulting in novel ∼1.8-kb *Glu-1Ax* alleles in some F_1_ and F_2_ plants. The cloned mutant *Glu-1Ax* alleles were expressed in *Escherichia coli*, and the HMW-GSs produced matched the novel HMW-GSs found in the hybrids. The differential changes between the endosperm and the plant of the same hybrids and the data of *E. coli* expression of the cloned deletion alleles both suggested that mitotic illegitimate recombination between two copies of a short repeat sequence had resulted in the deletions and thus the changed HMW-GS compositions. Our experiments have provided the first direct evidence to show that mitotic illegitimate recombination is a mechanism that produces novel phenotypes in wide hybrids.

## Introduction

Wide hybrids can have novel traits or greatly changed magnitudes of expression of existing traits that their parents do not have and cannot be simply explained by introgression alone. These phenomena have long been noticed [1]–[6], yet the mechanism is still poorly understood. Genomic rearrangement, transposition, expression change and epigenetic modification have been observed in artificial hybrids, double hybrids and recently formed allopolyploid species [7]–[12]. Genomic rearrangements caused by retrotransposon activation [13], homoeologous translocations between parental genomes [14], or gene expression changes [15]–[19] have been thought to be the causes. However, evidence establishing a causative link between these genetic/epigenetic changes and the novel phenotypic variations is still lacking.

High-molecular-weight glutenin subunits (HMW-GS) are storage proteins in the endosperm of wheat and its related species. They are encoded by complex *Glu-1* loci, which are located on homoeologous group 1 chromosomes. Each *Glu-1* locus consists of two paralogous genes of duplication origin that encode the x and y types of HMW-GSs, respectively. The two genes are separated by retrotransposon inserts [20]–[23]. However, their linkage is so tight that there has been no confirmed recombination between them [24]. In bread wheat, the *Glu-1* loci are on chromosomes 1A, 1B and 1D, and thus called *Glu-1A*, *Glu-1B* and *Glu-1D*, respectively. Although bread wheat has six HMW-GS alleles (i.e. *Glu-1Ax, Glu-1Ay, Glu-1Bx, Glu-1By, Glu-1Dx* and *Glu-1Dy*) but usually only three to five of them are simultaneously expressed due to gene silencing [25].

In this study, we elucidated the molecular mechanism that produces novel HMW-GSs in hybrids between bread wheat and rye. Changes in HMW-GS patterns of the hybrids and their derivatives were evaluated with a reference to changes in their coding alleles. The results presented here provide experimental evidences that establish a causative link between novel HMW-GS patterns and novel *Glu-1* alleles. We believe that short-homology-dependent illegitimate recombination events during mitosis in sporophytes were responsible for the observed changes in HMW-GS compositions. Therefore, mitotic illegitimate recombination should be considered as one of the molecular mechanisms that produce novel phenotypic variations in a wide hybrid.

## Results

### Novel HMW-GS patterns were observed in hybrid seeds

A total of 2,639 hybrid seeds between Japanese wheat landrace Shinchunaga and Chinese rye landrace Qinling were produced from 3124 pollinated florets without embryo rescue. The high seed-setting rate (84.47%), due to the high crossability genes carried by Shinchunaga [26], enabled us to work with a large F_1_ population without the risk of mutant induction by embryo rescue procedure. Of these F_1_ seeds, 2078 had sufficiently plump endosperm to be selected for HMW-GS analysis.

**Figure 1:**
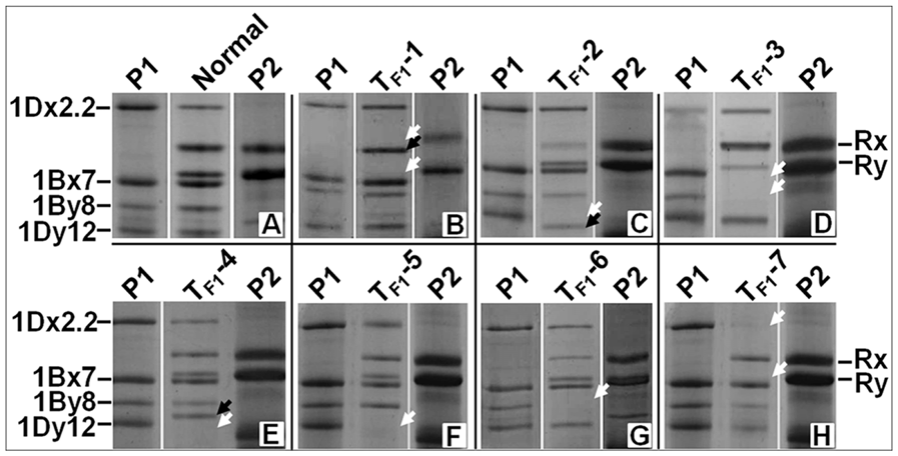
Observed HMW-GS changes (T_F1_-1–T _F1_-7) in F_1_ hybrid seeds. P1: Shinchunaga wheat; P2: Qinling rye. White arrow head: position of an absent subunit; black arrow head: a novel subunit.

*Glu-1* alleles are co-dominant. Every functional allele encodes a distinguishable HMW-GS. In Shinchunaga, the *Glu-1Ax* and *Glu-1Ay* genes are null. As shown in Figure 1a, Shinchunaga has subunits Bx7+By8 and Dx2.2+Dy12, and Qinling has subunits Rx+Ry. Therefore, the F_1_ hybrid seeds should have the combined subunits of their parents, i.e., Bx7+By8, Dx2.2+Dy12 and Rx+Ry. That was exactly what we observed in 99.5% of the F_1_ seeds we analyzed. Interestingly, we also observed seven new HMW-GS patterns (Figure 1, Table 1) in ten F_1_ seeds, each from a different spike. For instance, a new HMW-GS subunit appeared, and the two rye subunits disappeared in four of the ten F_1_ seeds (T_F1_-1, Figure 1b). Other new patterns either have a smaller or larger 1Dy12 variants or lack one or two subunits (Figure 1c–1h, Table 1). The occurrence of variants, 0.5%, was too low to suggest that the new patterns were due to heterozygosity of the parents. Some *de novo* changes must be responsible for these variations. Unfortunately, only three seedlings were obtained and they all died within one week after germination, so none of the ten F_1_ seeds was able to produce a vigorously growing an F_1_ plant for further investigation.

**Table 1:** HMW-GS variations in F_1_ wheat-rye hybrid seeds.

Code number for hyhrid spike	No. of F_1_ seeds analyzed	No. of varied seed	Variation type
A-2	23	1	T_F1_-1 (Figure 1b)
A-44	43	1	T_F1_-1
A-46	22	1	T_F1_-1
A-53	23	1	T_F1_-1
B-2	22	1	T_F1_-2 (Figure 1c)
E-3	14	1	T_F1_-3 (Figure 1d)
J-1	18	1	T_F1_-4 (Figure 1e)
M-2	27	1	T_F1_-5 (Figure 1f)
A-0	21	1	T_F1_-6 (Figure 1g)
A-65	24	1	T_F1_-7 (Figure 1h)

**Figure 2:**
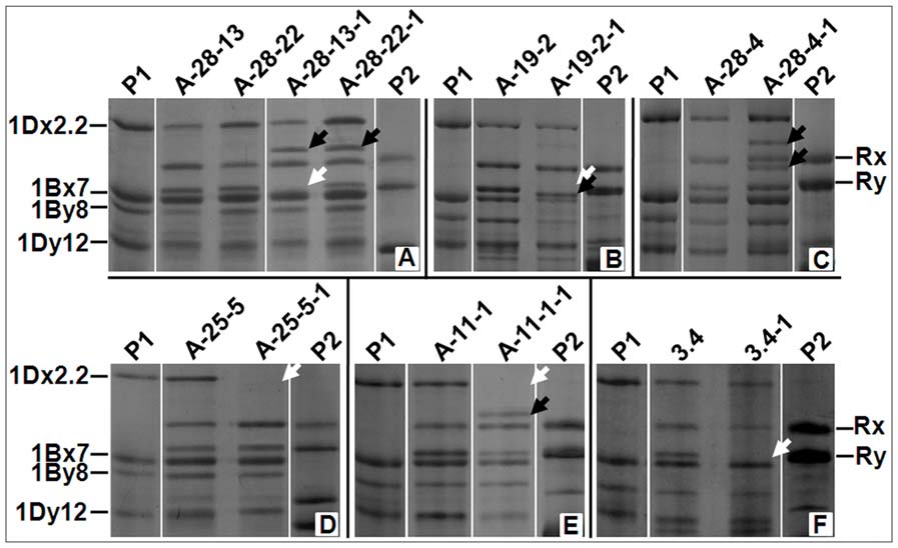
Examples of HMW-GS changes in the normal F_1_-derived F_2_ seeds. P1: Shinchunaga wheat; P2: Qinling rye. White arrow head: position of an absent subunit; black arrow head: a novel subunit.

Of the F_1_ seeds that had the wildtype HMW-GS pattern, 707 were randomly selected for germination and 136 vigorously growing F_1_ plants were obtained. Most of the F_1_ plants were sterile and only 26 were able to produce a total of 36 F_2_ seeds. These 36 F_2_ seeds were studied for their HMW-GSs. Our results showed that eight of them had new HMW-GS patterns, while the rest had the wildtype (Table 2). As we observed in the F_1_ seeds, variations of the HMW-GS patterns in the F_2_ seeds included both losses of expected subunits and/or additional novel subunits (Figure 2). However, the novel types all different from what was seen in the F_1_ seeds (Figure 2). Since the F_1_ parents of the eight F_2_ seeds had only the wildtype HMW-GSs, the new patterns in the F_2_s seemed to occur via *de novo* changes.

**Table 2:** HMW-GS variations in selfed F_2_ wheat-rye hybrid seeds.

Code no. of F1 plant	No. of F2 seeds	No. of varied seeds (seed code number)	Variation type
A-28-13	1	1 (A-28-13-1)*	A new subunit appeared (Figure 2a).
A-28-22	1	1 (A-28-22-1)*	A new subunit appeared and Ry disappeared (Figure 2a).
A-19-2	2	1 (A-19-2-1)**	Ry disappeared and a new subunit appeared (Figure 2b).
A-28-4	4	1 (A-28-4-1)**	Two new subunits appeared (Figure 2c).
A-25-15	1	1 (A-25-15-1)	Subunit 2.2 disappeared (Figure 2d).
A-11-1	1	1 (A-11-1-1)	Subunit 2.2 disappeared and a new subunit appeared (Figure 2e).
3.4	1	1 (3.4-1)**	Subunit Ry disappeared (Figure 2f).
14.3-4	1	1 (14.3-4-1)**	Subunit Ry disappeared.
0ther 18 F1 plants	24	0	

*Mature F_2_ plants were successfully obtained from seeds;

**F_3_ seeds were successfully obtained by selfing the F_2_ plants.

Six of the eight F_2_ seeds that showed new HMW-GS patterns were able to grow into mature F_2_ plants and four of them set some F_3_ seeds (Table 2). Meanwhile, 15 F_2_ plants were obtained from the 28 F_2_ seeds that showed the wildtype HMW-GS pattern. Of the 15 F_2_ plants, seven were able to set plenty of F_3_ seeds. Obviously, the survival rate of the seeds with altered HMW-GSs was greatly improved in the F_2_s (75% in the F_2_s vs. 0% in the F_1_s).

### Deletions lead to the *de nova* changes in HMW-GS subunits

**Figure 3:**
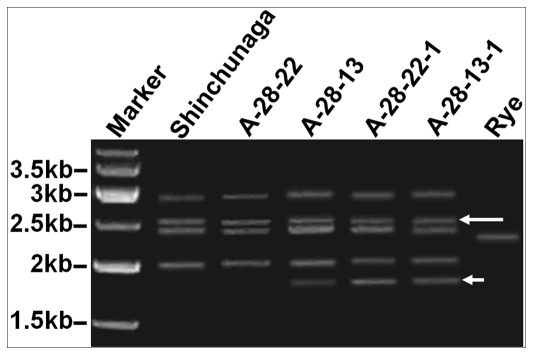
PCR amplification of HMW-GS genes in hybrids and their parent plants. A novel fragment about 1.8 kb (short arrowhead) was revealed in F_1_ plant A-28-13, F_2_ plants A-28-13-1 and A-28-22-1. The about 2500-bp fragments of *1Ax* null gene existed in all hybrid plants and wheat parent (long arrowhead).

To understand the molecular mechanism of the observed *de nova* changes in HMW-GSs in the hybrid seeds, we investigated changes in their coding sequences in the genomes of the F_1_ and F_2_ plants derived from these hybrid seeds. The coding sequences were amplified by PCR out of the genomic DNA of Shinchunaga, Qinling, their F_1_ plants A-28-13 and A-28-22, and the F_2_ plants A-28-13-1 and A-28-22-1, with a pair of universal primers for HMW-GS genes (Figure 3). The two F_2_ plants were chosen because they both showed altered HMW-GSs in their endosperms but both their F_1_ parents A-28-13 and A-28-22 only had the wildtype subunits in their endosperms (Figure 2a). As Figure 3 showed, our PCR assays revealed four PCR bands in Shinchunaga and one in Qinling. The universal primers failed to amplify the band from Qinling in the hybrids although our protein assay had demonstrated that the rye genes existed in the hybrid seeds (Figure 2). The failure was possibly due to template competition in PCR. Interestingly, a ∼1.8-kb PCR band appeared in all the hybrids but the F_1_ plant A-28-22 (Figure 3). All of the PCR products were cut from the gels and cloned for further analyses.

Sequence analyses of the cloned PCR products indicated that they all matched the expected *Glu-1* genes, and all but the ∼1.8-kb fragments were of wildtype. Interestingly, the ∼2.5-kb fragment shown on Figure 3 was actually the silenced *Glu-1Ax* allele and the ∼1.8-kb fragments were all derivatives from this wildtype *Glu-1Ax* null allele.

**Figure 4:**
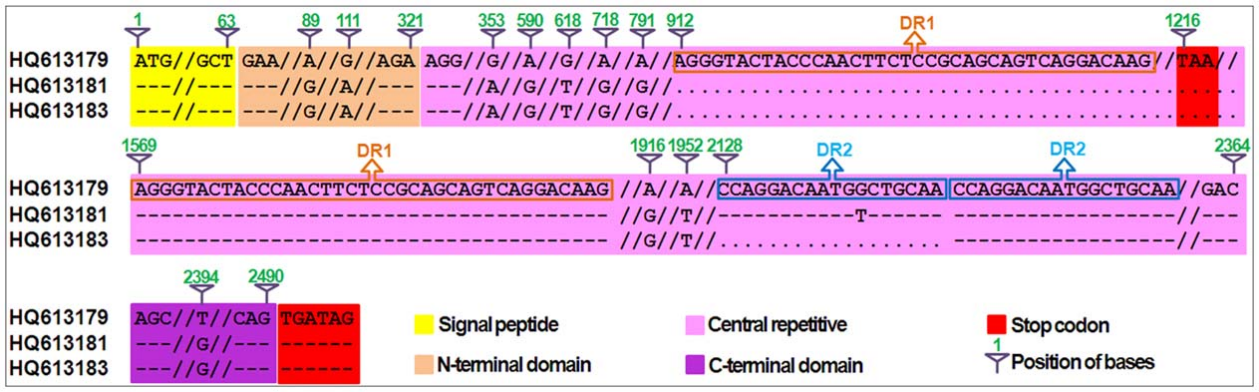
A sequence comparison between the wildtype *Glu-1Ax* (HQ613179) and two deletion mutations DM1 (HQ613181) and DM2 (HQ613183) in F_1_ plant A-28-13. Deleted nucleotides were indicated as “.” and identical nucleotide sequences were shown as “–”. DR1: the type 1 direct repeat sequence type 1; DR2: the type 2 direct repeat sequence. The numbers above the sequence indicate the positions in the wildtype.

As illustrated in Figure 4, the wildtype *Glu-1Ax* null allele (HQ613179) cloned from Shinchunaga is 2496 bp long and contains a signal peptide (positions 1–63 bp), an N-terminal region (64–321 bp), a central repetitive region (322–2364 bp) that consists of short repeat sequences, and a C-terminal region (2365–2490 bp). An in-frame stop codon (TAA) at the 1216–1218-bp position in the central repetitive region seems to have prematurely attenuated the translation of 1Ax subunits in Shinchunaga. Deletion of this in-frame stop codon could lead to reactivation of the *Glu-1Ax* null allele.

Twenty clones of the ∼1.8-kb fragments from the F_1_ plant A-28-13 were sequenced and compared with the wildtype *Glu-1Ax* null allele (HQ613179) (Figure 4). Apparently, these clones were a mixture of two types (HQ613181 and HQ613183) of deletion mutant (DM) alleles (DM1 and DM2) from the wildtype. DM1 apparently resulted from a 657-bp deletion at the 912–1568-bp position in the central repetitive region plus 11 SNPs, while DM2 had an additional 18-bp deletion in this region. Of the 20 clones from the F_1_ plant A-28-13, only HQ613183 had DM2, while the remaining 19 all had DM1.

To study the ∼1.8-kb fragments from the F_2_ plants, 33 clones from the F_2_ plant A-28-13-1 and 37 from the F_2_ plant A-28-22-1 were sequenced and analyzed. From these 70 clones, a total of 52 different sequence types, ranging from 1659 to 1884 bp in length, were obtained and all were found to be derived from the wildtype *Glu-1Ax* null allele via one or two deletions of 180–774 bp in length in the central repetitive region plus some SNPs (Table S1). Of the 70 clones, 18 clones (10 from A-28-13-1 and 8 from A-28-22-1) had DM1 but none had DM2. In addition, 39 new DMs were observed in the F_2_ plants. DM17 was observed in five and four clones from A-28-13-1 and A-28-22-1, respectively. Other DM alleles existed only in one or two clones.

All of the deletions observed in the wheat-rye hybrids were found to start and end at a direct repeat sequence (DR) unit in the central repetitive region. For example, the starting (912 bp) and ending (1568 bp) positions of DM1 were exactly where two 38-bp DR1 units located (Figure 4). A total of 42 DR types, each was of 3 to 60 bp per unit, were observed in the deletions. The number of repeat units per DR varied from 2 to 100 (Table S1). The length of a DR was significantly negatively correlated with the times it repeated (r = −0.52, p≤0.01). For instance, DR31 has 4 bp per unit and repeats 100 times in the wildtype *Glu-1Ax* allele; meanwhile DR17 has 60 bp per unit and only repeats twice. Apparently, there also was a significantly positive correlation (r = 0.68, p≤0.01) between DR length and the frequency that it is involved in the deletions.

**Figure 5:**
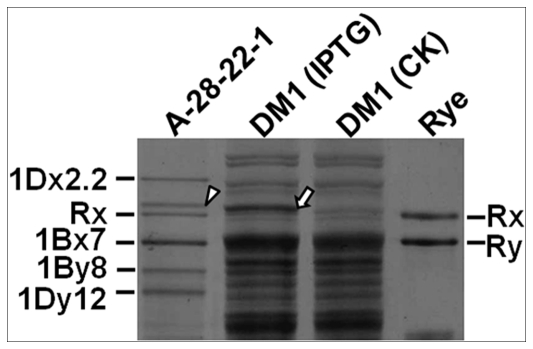
An example of bacterial expression of a new allele (GenBank number HQ613181) with deletion mutation type 1 (DM1). IPTG: *E. coli* culture with isopropyl β-Δ-thiogalactopyranoside; CK: *E. coli* culture without IPTG; arrowhead: the new HMW subunit extracted from F_2_ seed A-28-22-1; tailed arrowhead: the expressed mature protein from *E. coli* culture.

**Figure 6:**
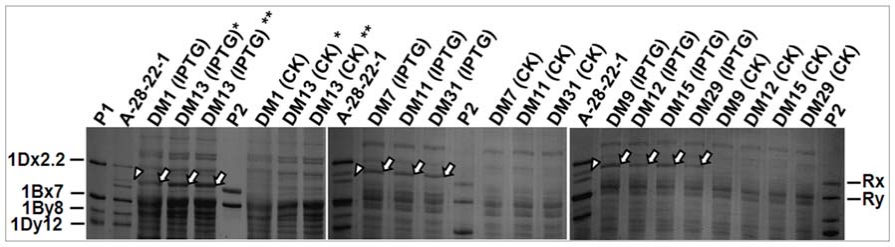
Bacterial expression of some deletion mutations (DM) from F_2_ plants. P1: Shinchunaga wheat; P2: Qinling rye; IPTG: *E. coli* culture with isopropyl β-Δ-thiogalactopyranoside; CK: *E. coli* culture without IPTG; arrowhead: the new HMW subunit extracted from F_2_ seed A-28-22-1; tailed arrowhead: the expressed mature protein from *E. coli* culture. The sequences used for expression were HQ613181 (DM1), HQ613197 (DM13*), HQ613222 (DM13**), HQ613188 (DM7), HQ613195 (DM11), HQ613220 (DM31), HQ613190 (DM9), HQ613199 (DM15), HQ613196 (DM12), and HQ613218 (DM29).

Interestingly, the number of deleted nucleotides in all of the DMs was always the multiple of three, suggesting no frame-shift mutation in downstream. Therefore, each new allele, if it had the in-frame stop codon deleted and thus was active, could encode a novel *Glu-1Ax* subunit. In fact, as shown late in this section, we did successfully express some cloned new alleles (Figures 5 and 6) with an *Escherichia coli* expression system. For instance, DM8, DM10, DM11, DM12 and DM13 all have a deletion of the same length (738 bp) that contains the in-frame stop codon (Table S1), and they all should actively encode a novel 1Ax subunit (Figure 6). However, the deletions start and end at different positions. Therefore, the polypeptides they encode might be different and so are their molecular weights. The resolution of the SDS-PAGE system used in this study might not allow us to differentiate all of the novel HMW-GS subunits since they might have similar molecular weights or migration mobility but differ slightly in amino acid compositions (Figure 6).

### *E. coli* expression of a novel 1.8-kb *Glu-1Ax* allele that makes a new 1Ax subunit

To verify that the observed ∼1.8-kb fragments indeed are novel *Glu-1Ax* alleles, a modified 1.8-kb fragment with DM1 (GenBank accession HQ613181) was cloned from the F_1_ plant A-28-13 and inserted into expression vector pET-30a, which was then transformed into *E. coli* for an expression test. The modification was done by removing the signal peptides. Protein samples from the culture of the transformed *E. coli* with and without inducer IPTG were assayed by SDS-PAGE together with the protein samples from the F_2_ seed A-28-22-1 and Qinling (Figure 5). This assay showed that the 1.8-kb fragment from the F_1_ plant A-28-13 encoded a protein similar in MW with the speculated novel 1Ax subunit observed in the endosperm of the F_2_ seed A-28-22-1 (Figure 5) and A-28-13-1 (Figure 2a). Some other novel alleles were expressed with this *E. coli* expressing system as well (Figure 6). These results, therefore, confirmed our hypothesis that a novel *Glu-1Ax* allele (like the one with DM1) could result from a deletion of the fragment containing the in-frame stop codon from the wildtype *Glu-1Ax* null allele (like the one in the F_1_ plant A-28-13) and it will actively express a new 1Ax subunit (like the one in the F_2_ seeds A-28-13-1 and A-28-22-1).

## Discussion

### Novel HMW-GSs might result from illegitimate recombination between direct repeat sequences

Our data showed that some novel HMW-GS patterns emerged in hybrids (Figures 1 and 2). Our first question was where they came from. Our observation showed that: 1) only 0.05% of the 2,078 F_1_ seeds analyzed had a novel HMW-GS pattern; 2) novel patterns appeared in the F_2_ seeds that was not seen in their F_1_ parents; 3) 42 types of short repeat sequences exist in *Glu-1Ax* gene that were involved in deletion; 4) all of the observed deletions in the *Glu-1Ax* gene were flanked by a DR unit; and 5) each deletion event apparently removed a DR unit from the sequence in addition to the deleted fragment. Previous studies [27]–[29] have indicated that the presence of flanking DR units is typical evidence or a “signature”, as Wicker et al. [30] suggested, of an illegitimate recombinant event. It seemed that the observed novel *Glu-1Ax* alleles were the result of illegitimate recombination events, and at least the observed deletions most likely occurred between the DR units in the central repetitive region of the *Glu-1Ax* null allele. All of the observed deletions in the hybrids seemed to occur *de novo* though DM1 could also be transmitted from the F_1_ to the F_2_ generation.

**Figure 7:**
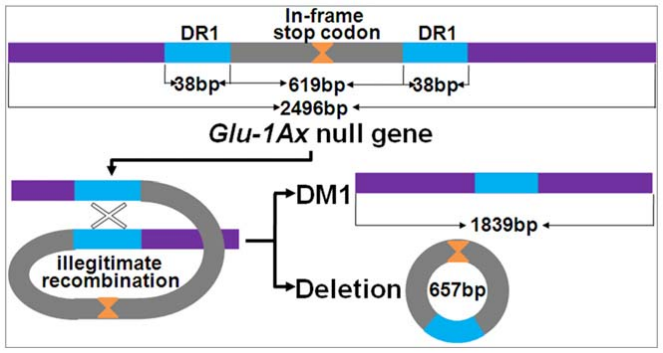
The proposed origin of DM1 via illegitimate recombination between two copies of the type 1 direct repeated sequence (DR1).

Figure 7 illustrated such an illegitimate recombination event we speculated with specific reference to the origin of DM1. It showed that an illegitimate recombination between two DR1 units apparently led to a 657-bp deletion that consists of the in-frame stop codon and hence made a new active allele (HQ613181) of 1839 bp in length from the wildtype *Glu-1Ax* null (Figure 4). Indeed, *E. coli* that expressed this new allele did produce the same novel 1Ax subunit as observed in the endosperm of the F_2_ seed A-28-22-1 (Figure 5) and A-28-13-1 (Figure 2a).

We observed that some HMW-GSs were missing in some F_1_ and F_2_ seeds examined. Since each *Glu-1* locus contains two tightly linked paralogous genes for the x and y types of HMW-GS proteins, missing their carrier chromosome will lead to loss of the locus and thus both HMW-GSs in F_2_ seeds. However, we did not see this phenomenon in any F_2_ hybrid seed analyzed (Figure 2). Therefore, missing their carrier chromosomes should not be the cause of missing these HMW-GSs in the seeds. Considering that illegitimate recombination is able to create novel active alleles from the wildtype *Glu-1Ax* null via deletion, the same mechanism should also be able to produce a critical deletion that inactivates an active allele, resulting in missing a HMW-GS. Elucidating deletions in the endosperm that misses HMW-GSs should find the answer to this mystery.

### Illegitimate recombination most likely occurred during mitosis in sporophytes

**Figure 8:**
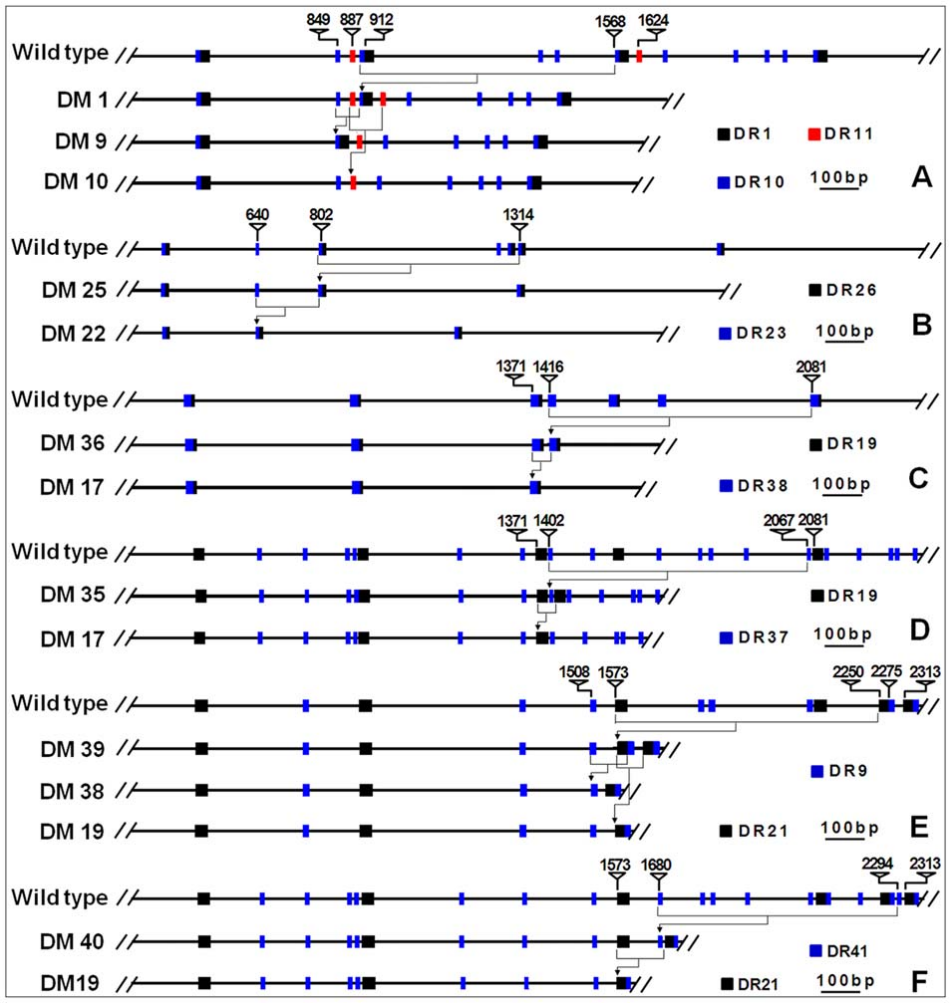
Illustrations of some secondary deletions via illegitimate recombination. DM9 and DM10 could come from secondary deletions of DM1 (A), DM22 from DM25 (B), DM17 from DM36 (C) or DM35 (D), DM19 and DM38 from DM39 (E), or DM19 from DM40 (F), respectively. DRs (direct repeat sequence type) nearby the deletion regions were indicated. Numbers above the sequence are the nucleotide positions in the wildtype.

Our second question was when the illegitimate recombination had taken place. Illegitimate recombination can take place during meiosis or mitosis. We know that the egg and the polar nuclei in the ovary of a hybrid are developed from a single macrospore and the two sperms that fertilize the egg and the polar nuclei are derived from a single microspore. In other words, the endosperm and the embryo (thus the plant developed from it) of a hybrid seed are genetically identical except that the endosperm has two copies of the maternal genome. If an illegitimate recombination takes place during meiosis, the resulting recombinants should exist in both the endosperm and the plant. If such a recombination is a mitotic event after the meiosis, it can only impact either the endosperm or the plant but not both. Our data showed that F_1_ A-28-13 had the wildtype HMW-GS pattern in its endosperm (Figure 2a) yet a 1.8-kb novel active allele in its leaves (Figure 3). We also observed that the novel HMW-GS compositions in the endosperms did not always match the types of mutant alleles in the plant grown up from the same hybrid seed and that there were more DM types in a hybrid plant than altered HMW-GSs in the endosperm of the same hybrid seed. All of these observations made us believe that the causative illegitimate recombination might have taken place after the double fertilization occurred. Therefore, what happened in the endosperm is irrelevant to what happened in the embryo. The speculation that the illegitimate recombination most likely occurred during mitosis in sporophytes instead of meiosis in gametophytes is also supported by our observation of co-existence of the wildtype *Glu-1Ax* null and the novel 1.8-kb *Glu-1Ax* alleles in the same F_1_ plant A-28-13, of which the genomes were in haploid status. In this case the illegitimate recombination might have only involved one sister chromatid as illustrated in Figure 7. Mitotic occurrence of illegitimate recombination could also explain the co-existence of both DM1 and DM2 in a single F_1_ plant and only DM1 was seen in the derived F_2_ plants. In this case, the illegitimate recombination that produced DM2 should have taken place sometimes after the mitosis that had produced DM1 in a copy of the DM1 allele. The second deletion most likely happened in a cell lineage that was not lead to germ line cells. In fact, some DMs we observed in the F_2_ plants might have been similarly produced via a two-step illegitimate recombination event. For instance, a further deletion in DM1 could have produced DM9 or DM10 allele (Table S1, Figure 8).

**Figure 9:**
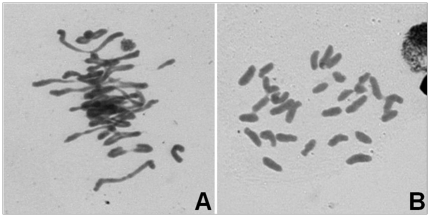
Examples of chromosome pairing at meiotic metaphse I. A: Most chromosomes are paired in a pollen-mother-cell of F_2_ plant A-28-13-1; B: All the chromosomes were unpaired in a pollen-mother-cell of F_1_ palnt A-28-13.

What happened in F_2_s was more complicated than in the F_1_s. This is due to the fact that the genomes in the F_2_s were more complex by having various numbers of homologous pairs (Figure 9a) than the simple haploid status in the F_1_s (Figure 9b). The new alleles observed in the F_2_s could be inherited from the F_1_ parent plants or be made *de novo* in the F_2_s. For example, the novel DM1*Glu-1Ax* allele created in the F_1_ plant A-28-13 seemed to be transmitted to the F_2_ A-28-13-1 as evidenced by the existence of this allele in the F_2_ plant A-28-13-1 (Figure 3) and by the appearance of the new *Glu-1Ax* allele in the endosperm of the F_2_ seed A-28-13-1 (Figure 2a). On the other hand, the F_1_ A-28-22 had the wildtype HMW-GS pattern in its endosperm (Figure 2) and only the wildtype *Glu-1Ax* null allele in its plant (Figure 3). Yet, the derived F_2_ A-28-22-1 had the novel 1Ax subunit in its endosperm (Figure 2) and had its coding DM1 *Glu-1Ax* allele (Figure 3, Table S1), indicating *de nova* mutations in both the F_2_ endosperm and plant respectively. It is difficult to explain the much higher deletion rate observed in the F_2_ population (Table S1) although it might result from the increased homologue in the genomes of the F_2_s that could lead to an increased illegitimate recombination rate since more *Glu*-*1A* gene-containing chromatids were available for more illegitimate recombination events.

### Mitotic illegitimate recombination is at least one of the mechanisms that product novel trait in hybrids and thus a factor of genome evolution in polyploids

Production of novel traits in wild hybrids is a mechanism that enhances genetic diversity. Nevertheless, as Khasdan et al. [31] reviewed, there have been only speculations of its molecular basis. No experimental evidence has been available so far. The present study has revealed that deletions caused by mitotic illegitimate recombination can lead to novel HMW-GSs, and thus provided the first direct evidence for the molecular mechanism.

Illegitimate recombination, like normal recombination, requires the action of enzymatic recombination complex. A possible molecular mechanism for illegitimate recombination to take place in hybrids, nevertheless, is that diverse homoeologous loci in the wide hybrids could direct the assembly of a novel recombination complex with diverse components, similar to the one proposed by Johnson and Heiss [32], in the hybrid nucleus, making normally impossible recombination between the short repeat sequences on the same chromosome possible. Alternatively, an siRNA-driven DNA-binding protein complex that normally protect these short repeat sequences from illegitimate recombination becomes less functional in a wide hybrid due to greater than normal genome diversity that brings in increasingly incompatible components. Perhaps this is the “genome shock” proposed by McClintock [13]. These hypotheses also fit the siRNA-mediated epigenetic mechanism speculated by Jackson and Chen [33] for genomic and expression plasticity in polyploids. Elucidating the recombination complex and differential actions by siRNA in the normal and the hybrid nuclei should make the picture clearer.

In summary, we simultaneously studied the endosperm and leaves of a wheat-rye hybrid and its derivatives for changes in their HMW-GS compositions and in the coding genes. Our study demonstrates that mitotic illegitimate recombination is a cause of novel phenotypes in the hybrids.

## Materials and Methods

### Ethics Statement

Plant materials used in this study included Japanese common wheat (*T. aestivum* L., 2n = 6× = 42, AABBDD) landrace Shinchunaga and Chinese rye (*Secal ecereale* L., 2n = 2× = 14, RR) landrace Qinling. Shinchunaga was provided by Dr. Shin Taketa of Barley Germplasm Center, Research Institute for Bioresources, Okayama University, Japan. Qinling came from the Germplasm Collections at Sichuan Agricultural University. It was originally collected from China by Prof Chi Yen and openly available in the public domain.

### Production of the wheat-rye hybrids

A cross was made between Shinchunaga and Qinling. Qinling had been selfed five times before being used. The emasculation and pollination were done as previously described [34]. Generally, spikes were emasculated after removing apical and basal spikelets and all but the two outermost florets from each of the remaining spikelets in order to achieve similar stages of maturity for entire spikes. Two or three spikes per Shinchunaga plant were emasculated and pollinated with freshly collected rye pollens two to three days after emasculation. The emasculated spikes were bagged before and after pollination to avoid pollination with other plants. No embryo rescuing or hormone treatment was applied. In about 30 days after pollination, the mature hybrid seeds were harvested separately by spikes and preserved for further study. Crossability was expressed as the percentage of the number of the F_1_ seeds obtained to the number of florets pollinated.

Hybrid seeds that were chosen for HMW-GS assay were cut in asymmetrically. The halves with the embryos were germinated in Petri dishes and the resulting seedlings were transplanted into an experimental plot 20 cm apart with 30 cm row spacing. Hybrid spikes were bagged before anthesis to prevent crossing contamination. F_2_ hybrid seeds were harvested from the selfed F_1_ plants. Chromosomes of the hybrids were observed according to Zhang et al. [26].

### SDS-PAGE analysis

The halves of the seeds that did not have the embryos were used to identify HMW-GS compositions. Protein samples were prepared from the endosperm and assayed by sodium dodecyl sulfate polyacrylamide-gel electrophoresis (SDS-PAGE) as described by Wan et al. [35]. Qinling has a HMW-GS composition of Rx+Ry on its *Glu-R1* locus, and the HMW-GS composition of Shinchunaga is null, 1Bx7+1By8, and 1Dx2.2+1Dy12 on *Glu-A1*, *Glu-B1* and *Glu-D1* loci, respectively [36].

### DNA preparation and PCR amplification

Leaf samples were collected at heading stage from the wheat and rye parents and their hybrids, ground in liquid nitrogen and used for DNA isolation with a modified CTAB method [37]. A pair of universal primers (5′-ATCACCCACAACACCGAGCA-3′; 5′-AGCTGCAGAGAGTTCTATCA-3′) was synthesized according to Xie et al. [38] and used to amplify the complete coding region of the *Glu-1* gene. PCR amplification was carried out in a PTC-200 thermocycler (MJ research). Each PCR reaction (50 µl) contained 200 ng of template DNA, 0.2 mM of each dNTPs, 1 µM of each of primer, 5 µl of 10×Ex Taq PCR buffer, 1.25 U of Ex*Taq* DNA polymerase with high fidelity (TaKaRa, Dalian, China) and ddH_2_O to 50 µl volume. The PCR reactions were programmed for 4 min at 94°C, followed by 25 cycles of 94°C for 40 s and then 68°C for 8 min. The final extension was kept for 8 min at 68°C. The PCR products were separated in 0.8% agrose gels.

### Cloning, sequencing and comparative analyses of the HMW-GS genes

The PCR products of interest were cut from the agrose gels and purified using E.Z.N.A.® Gel Extraction Kit (OMIGA). The DNA fragments were then ligated into the pMD18-T plasmid vector (TaKaRa, Dalian, China). The ligated products were transformed into competent cells of *E. coli* DH5α strain. A full-length sequence of each HMW-GS gene was obtained and confirmed by sequencing a set of subclones, which were made by the nested deletion methods [37]. Multiple sequence alignments were carried out using DNAMAN version 5.2.2.

DM types were determined based on the deletion position and length. DR types were based on the unique perfect direct repeat sequences. A single nucleotide polymorphism (SNP) was determined if a change nucleotide had appeared at that position in three or more clones.

### Expression of *Glu-1Ax* genes in *E. coli*

Modified open reading framess of 1Ax without the signal peptides were amplified from the cloned *Glu-1Ax* alleles by PCR using the primers: 5′-ACCCATATGGAAGGTGAGGCCTCTGGGC-3′ and 5′-TTCCTCGAGCTATCACTGGCTGGCCAAC-3′ (*Nde*I or *Xho*I restriction site underlined). The modified PCR products were cloned into the expression vector pET-30a (Novagen) and transformed into *E. coli* strain *BL21 (DE3) pLsS*. Expression of HMW-GSs in *E. coli* was induced by 1 mM isopropyl β-Δ-thiogalactopyranoside (IPTG) for 5 h. Protein samples were prepared from 1.5 ml of induced or un-induced bacterial cultures according to Wan et al. [39] and used for SDS–PAGE analysis.

## Supporting Information

Table S1 **The deletion mutation (DM) types observed in hybrids and direct repeats (DR) existed in the wildtype** ***Glu-1Ax*** **allele.**
(DOCX)
