# Supplementary material for: Mitotic Illegitimate Recombination Is a Mechanism for Novel Changes in High-Molecular-Weight Glutenin Subunits in Wheat-Rye Hybrids
Source: PLoS One. 2011 Aug 22;6(8):e23511. doi: 10.1371/journal.pone.0023511 (PMC3161740; doi:10.1371/journal.pone.0023511)
Supplement: Table S1 — The deletion mutation (DM) types observed in hybrids and direct repeats (DR) existed in the wildtype Glu-1Ax allele. (DOCX) [file pone.0023511.s001.docx]

**Table S1.** The deletion mutation (DM) types observed in hybrids and direct repeats (DR) existed in the wildtype *Glu-1Ax* allele.

| DM type (no. of clones) | GenBank Accession no. | Sequence size in bp | Deletion position  (size in bp) | DR type  (size in bp) | No. of repeats of a DR |
| --- | --- | --- | --- | --- | --- |
|  |  |  |  |  |  |
| **Hybrid F_1_ plant A-28-13** | | | | | |
| DM 1 (16) | HQ613181 | 1839 | 912-1568 (657) | DR1 (38) | 4 |
| DM 1 (3) | HQ613182 | 1839 | 912-1568 (657) | DR1 (38) | 4 |
| DM 2 (1) | HQ613183 | 1821 | 912-1568 (657) | DR1 (38) | 4 |
|  |  |  | 2128-2145 (18) | DR2 (18) | 2 |
| **Hybrid F_2_ plant** **A-28-13-1** | | | | | |
| DM 1 (4) | HQ613181 | 1839 | 912-1568 (657) | DR1 (38) | 4 |
| DM 1 (1) | HQ613194 | 1839 | 912-1568 (657) | DR1 (38) | 4 |
| DM 1 (3) | HQ613192 | 1839 | 912-1568 (657) | DR1 (38) | 4 |
| DM 1 (2) | HQ613193 | 1839 | 912-1568 (657) | DR1 (38) | 4 |
| DM 3 (1) | HQ613184 | 1785 | 392-1102 (711) | DR3 (10) | 26 |
| DM 4 (1) | HQ613185 | 1848 | 451-1098 (648) | DR4 (12) | 14 |
| DM 5 (1) | HQ613186 | 1848 | 488-1135 (648) | DR5 (11) | 9 |
| DM 6 (1) | HQ613187 | 1803 | 491-1183 (693) | DR6 (26) | 6 |
| DM 7 (1) | HQ613188 | 1884 | 558-980 (423) | DR7 (8) | 17 |
|  |  |  | 1168-1356 (189) | DR8 (11) | 27 |
| DM 8 (1) | HQ613189 | 1758 | 770-1507 (738) | DR9 (17) | 8 |
| DM 9 (1) | HQ613190 | 1776 | 849-1568 (720) | DR10 (14) | 11 |
| DM 10 (1) | HQ613191 | 1758 | 887-1624 (738) | DR11 (16) | 2 |
| DM 11 (1) | HQ613195 | 1758 | 917-1654 (738) | DR12 (17) | 9 |
| DM 12 (1) | HQ613196 | 1758 | 1062-1799 (738) | DR13 (13) | 4 |
| DM 13 (1) | HQ613197 | 1758 | 1103-1840 (738) | DR14 (19) | 3 |
| DM 14 (1) | HQ613198 | 1830 | 1130-1795 (666) | DR15 (4) | 47 |
| DM 15 (1) | HQ613199 | 1722 | 1184-1957 (774) | DR16 (16) | 11 |
| DM 16 (1) | HQ613200 | 1746 | 1341-1538 (198) | DR17 (60) | 2 |
|  |  |  | 1787-2338 (552) | DR18 (19) | 6 |
| DM 17 (2) | HQ613201 | 1785 | 1371-2081 (711) | DR19 (29) | 5 |
| DM 17 (1) | HQ613203 | 1785 | 1371-2081 (711) | DR19 (29) | 5 |
| DM 17 (2) | HQ613202 | 1785 | 1371-2081 (711) | DR19 (29) | 5 |
| DM 18 (1) | HQ613204 | 1830 | 1476-2141 (666) | DR20 (13) | 7 |
| DM 19 (1) | HQ613205 | 1755 | 1573-2313 (741) | DR21 (34) | 6 |
| DM 20 (2) | HQ613206 | 1836 | 1655-2314 (660) | DR12 (17) |  |
| **Hybrid F_2_ plant A-28-22-1** | | | | | |
| DM 1 (3) | HQ613181 | 1839 | 912-1568 (657) | DR1 (38) | 4 |
| DM 1 (1) | HQ613215 | 1839 | 912-1568 (657) | DR1 (38) | 4 |
| DM 1 (1) | HQ613216 | 1839 | 912-1568 (657) | DR1 (38) | 4 |
| DM 1 (1) | HQ613192 | 1839 | 912-1568 (657) | DR1 (38) | 4 |
| DM 1 (2) | HQ613193 | 1839 | 912-1568 (657) | DR1 (38) | 4 |
| DM 11 (1) | HQ613217 | 1758 | 917-1654 (738) | DR12 (17) | 9 |
| DM 13 (1) | HQ613222 | 1758 | 1103-1840 (738) | DR14 (19) | 3 |
| DM 17 (1) | HQ613201 | 1785 | 1371-2081 (711) | DR19 (29) | 5 |
| DM 17 (1) | HQ613202 | 1785 | 1371-2081 (711) | DR19 (29) | 5 |
| DM 17 (1) | HQ613225 | 1785 | 1371-2081 (711) | DR19 (29) | 5 |
| DM 17 (1) | HQ613226 | 1785 | 1371-2081 (711) | DR19 (29) | 5 |
| DM 19 (1) | HQ613232 | 1755 | 1573-2313 (741) | DR21 (34) | 5 |
| DM 21 (1) | HQ613207 | 1785 | 456-1166 (711) | DR22 (7) | 51 |
| DM 22 (1) | HQ613208 | 1821 | 640-1314 (675) | DR23 (12) | 7 |
| DM 23 (1) | HQ613209 | 1821 | 673-915 (243) | DR24 (5) | 21 |
|  |  |  | 1062-1493 (432) | DR13 (13) | 4 |
| DM 24 (1) | HQ613210 | 1749 | 679-1425 (747) | DR25 (9) | 17 |
| DM 25 (1) | HQ613211 | 1785 | 802-1314 (513) | DR26 (21) | 5 |
|  |  |  | 1351-1548 (198) | DR27 (50) | 2 |
| DM 26 (1) | HQ613212 | 1776 | 811-1530 (720) | DR28 (12) | 14 |
| DM 27 (1) | HQ613213 | 1839 | 898-1554 (657) | DR8 (11) | 27 |
| DM 28 (1) | HQ613214 | 1758 | 912-1370 (459) | DR19 (29) | 5 |
|  |  |  | 1507-1785 (279) | DR29 (41) | 2 |
| DM 29 (1) | HQ613218 | 1659 | 919-1575 (657) | DR30 (31) | 6 |
|  |  |  | 1771-1950 (180) | DR31 (4) | 100 |
| DM 30 (1) | HQ613219 | 1758 | 941-1678 (738) | DR3 (10) | 26 |
| DM 31 (1) | HQ613220 | 1758 | 1095-1832 (738) | DR32 (7) | 9 |
| DM 32 (1) | HQ613221 | 1755 | 1096-1590 (495) | DR33 (16) | 9 |
|  |  |  | 2022-2267 (246) | DR34 (6) | 25 |
| DM 33 (1) | HQ613223 | 1857 | 1300-1938 (639) | DR35 (11) | 14 |
| DM 34 (1) | HQ613224 | 1776 | 1337-2056 (720) | DR36 (8) | 19 |
| DM 35 (1) | HQ613227 | 1830 | 1402-2067 (666) | DR37 (13) | 18 |
| DM 36 (2) | HQ613228 | 1830 | 1416-2081 (666) | DR38 (21) | 7 |
| DM 37 (1) | HQ613229 | 1818 | 1491-1796 (306) | DR39 (39) | 2 |
|  |  |  | 1896-2267 (372) | DR40 (24) | 4 |
| DM 38 (1) | HQ613230 | 1728 | 1508-2275 (768) | DR9 (17) | 8 |
| DM 39 (1) | HQ613231 | 1818 | 1573-2250 (678) | DR21 (34) | 5 |
| DM 40 (1) | HQ613233 | 1881 | 1680-2294 (615) | DR41 (14) | 17 |
| DM 41 (1) | HQ613234 | 1836 | 1699-2358 (660) | DR42 (3) | 42 |

* DM: type of deletion mutant; DR: types of direct repeat sequences.
